# Amifostine reduces the seminiferous epithelium damage in doxorubicin-treated prepubertal rats without improving the fertility status

**DOI:** 10.1186/1477-7827-8-3

**Published:** 2010-01-10

**Authors:** Vanessa Vendramini, Estela Sasso-Cerri, Sandra M Miraglia

**Affiliations:** 1 Developmental Biology Laboratory, Department of Morphology and Genetics, Federal University of São Paulo (UNIFESP), São Paulo-SP, Brazil; 2 Laboratory of Histology and Embryology, Department of Morphology, Dental School of São Paulo State University (UNESP), Araraquara-SP, Brazil

## Abstract

**Background:**

Amifostine is an efficient cytoprotector against toxicity caused by some chemotherapeutic drugs. Doxorubicin, a potent anticancer anthracycline, is known to produce spermatogenic damage even in low doses. Although some studies have suggested that amifostine does not confer protection to doxorubicin-induced testicular damage, schedules and age of treatment have different approach depending on the protocol. Thus, we proposed to investigate the potential cytoprotective action of amifostine against the damage provoked by doxorubicin to prepubertal rat testes (30-day-old) by assessing some macro and microscopic morphometric parameters 15, 30 and 60 days after the treatment; for fertility evaluation, quantitative analyses of sperm parameters and reproductive competence in the adult phase were also carried out.

**Methods:**

Thirty-day-old male rats were distributed into four groups: Doxorubicin (5 mg/kg), Amifostine (400 mg/kg), Amifostine/Doxorubicin (amifostine 15 minutes before doxorubicin) and Sham Control (0.9% saline solution). "Standard One Way Anova" parametric and "Anova on Ranks" non-parametric tests were applied according to the behavior of the obtained data; significant differences were considered when p < 0.05.

**Results:**

The rats killed 30 and 60 days after doxorubicin treatment showed diminution of seminiferous epithelium height and reduction on the frequency of tubular sections containing at least one type of differentiated spermatogonia; reduction of sperm concentration and motility and an increase of sperm anomalous forms where observed in doxorubicin-treated animals. All these parameters were improved in the Amifostine/Doxorubicin group only when compared to Doxorubicin group. Such reduction, however, still remained below the values obtained from the Sham Control group. Nevertheless, the reproductive competence of doxorubicin-treated rats was not improved by amifostine pre-administration.

**Conclusions:**

These results suggest that amifostine promotes a significant reduction of the doxorubicin long-term side effects on the seminiferous epithelium of prepubertal rats, which is reflected in the epidydimal fluid parameters in the adult phase. However, fertility status results suggest that such protection may not be effective against sperm DNA content damage. Further investigation of sperm DNA integrity must be carried out using amifostine and doxorubicin-treated experimental models.

## Background

A perfect chemotherapeutic treatment would selectively attack tumor cells without causing toxicity on normal tissues. Unfortunately, this ideal selectivity has not yet been reached by traditional chemotherapy, which is known to affect both neoplastic and proliferating normal cells [1]. Modern therapies using multiple combinations of chemotherapeutic drugs reduce the cytotoxicity of these drugs to normal tissues, increasing the survival rates [2]. However, even after increasing the effectiveness of these treatments, many patients present post-chemotherapy sterility for about 5 years [3-5]. Besides, children and young patients exposed to chemotherapy in prepubertal phase can yet show irreversible impairment or loss of fertility status [6].

Among various antineoplastic agents, doxorubicin, an anthracycline compound, is one of the most used anticancer drugs. Doxorubicin has recognized effectiveness against solid and non-solid malignant tumors and is used in oncology protocols against malignancies such as Hodgkin disease, childhood leukemia and testicular cancer, which commonly affect young patients and children [3,6]. Nonetheless, it is responsible for long and short-term male infertility [7,8]. The preferential target of doxorubicin is the DNA of dividing cells; the drug intercalates within DNA strands causing cell cycle blockage in the G_2 _phase, single-strand breaks [9] and inhibition of the activity of some nuclear proteins, such as DNA and RNA-polimerase and DNA-topoisomerase II [10]. It has been recently found that doxorubicin also interferes with an important molecule involved in chromosome stability and transcription, the DNA methyl-transferase 1 - DNMT1 [11], inducing apoptosis.

Clinical and experimental studies have widely demonstrated the testicular toxicity caused by doxorubicin [12,13]. Lu and Meistrich [14] showed that even a low dose of doxorubicin (1 mg/kgb.w.) given to adult mice is able to target germ cells, mainly A1-A4 spermatogonia, leading to seminiferous epithelium depletion. Moreover, doxorubicin can also harm type B spermatogonia [15] and primary spermatocytes depending on the treatment schedule [14].

The fertility preservation of young patients submitted to anticancer treatments is an important aspect that must be considered, since the prognosis of 10-year survival after childhood leukemia has the projection to reach 90% until the end of 2010 [3,4]. Thus, the chemotherapy schedules also need to be improved; on this scope, other supporting therapies must be investigated focusing on reducing undesirable effects and providing a better life quality to survivor patients. Amifostine, a cellular protector, has been additionally used in chemotherapy and radiotherapy with this purpose [5].

Amifostine (WR-2721) is an organic phosphorylated thyol, which was isolated by the US Army in the 1950's, aiming to protect soldiers against a possible nuclear war. Initial clinical trials were focused on the prevention of hematotoxicities produced by radiation, cyclophosphamide, carboplatin and cisplatin therapies [16-18]. Besides the remarkable cytoprotection of amifostine against toxicities and side effects provoked by chemotherapeutic drugs, performed by its dephosphorylated metabolite, the WR-1065 free thyol, it also exhibits selective protection to normal cells without reducing the antitumor drug effectiveness. This selectivity is a consequence of some mechanisms that ease the capture of WR-1065 in normal tissues, which are much more vascularized than tumoral tissues [19,20]. Moreover, in non-tumoral tissues the alkaline phosphatase has the favorable neutral pH for its adequate activity, required to dephosphorilate the WR2721 (amifostine) to the active metabolite, WR-1065 [19-21]. This metabolite acts as ROS scavenger and stabilizes intact DNA inside normal cell nucleus, inhibiting DNA intercalation and breakage caused by antineoplastic drug [22]; such stability also improves the DNA ability to self-repair after any DNA damage that might have occurred after antitumoral treatments [21].

Although WR-2721 is being included in anticancer treatment schedules for both radiotherapy [5] and chemotherapy [23], there is scarce information about its chemical interactions and its systemic effects, especially in young patients and children. Although some works have been published presenting *in vitro *and *in vivo *results after exposure of young rats to both drugs [24-28], there are no detailed morphological studies concerning the potential protection conferred by amifostine to the seminiferous epithelium integrity and its recovery capacity. Moreover, since many children and adolescents have already been submitted to amifostine treatment prior to chemotherapy protocols including doxorubicin [5], the extension of the effects on male reproduction still need to be answered.

Until the present moment, there is no often information related to the fertility of patients who were simultaneously exposed to doxorubicin and amifostine during childhood and adolescence. Besides, previous findings by our group indicated that amifostine partially protects germ cells of prepubertal rats against apoptosis caused by cisplatin, another chemotherapeutic drug [24]. Thus, considering the wide use of doxorubicin against malignant neoplasm and the fact that infertility has become a common consequence of these treatments, we decided to investigate whether acute and previous administration of amifostine to prepubertal rats can protect their seminiferous epithelium against the doxorubicin acute treatment. For that reason, during the sexual maturation of the rats (prepubertal, pubertal and young adult phases), the impact of these treatments on the frequency of germ cell types and on testicular stereological and morphometric parameters was scrutinized. Adult fertility was also analyzed.

## Methods

### Animals and groups

Sixty 30-day-old male Wistar rats were distributed into four groups: Doxorubicin (D), Amifostine (A), Amifostine/Doxorubicin (AD) and Sham Control (SC). The animals were killed at 3 different times after treatment (15, 30 and 60 days after it), subdividing all the groups into 3 subgroups according to the killing ages (45-, 60- and 90-day-old rats). Thus, 12 subgroups of 5 animals each were established. The number indicated in each subgroup acronym corresponds to the sacrifice age. Additional males were included in each ninety-day-old group (totaling 10 males per group) to evaluate their reproductive competence. Rats were maintained under 12/12 hr light/dark cycles, at 21-23°C room temperature; standardized lab chow (Nuvilab CR1, Nuvital^®^, Curitiba, PR, Brazil) and water were provided *ad libitum*. This study was approved by the Ethical Committee for Animal Research of the Federal University of São Paulo, Brazil.

### Protocols of treatment

The experimental and control groups were the following: Sham Control group (SC), treated with 0.5 ml of 0.9% saline solution; Amifostine group (A) that received 400 mg/kg of amifostine ("Ethyol" or WR-2721; Schering-Plough S/A, São Paulo, Brazil); Doxorubicin group (D), treated with 5 mg/kg of doxorubicin (Eurofarma, São Paulo, Brazil); Amifostine/Doxorubicin group (AD), treated with amifostine (400 mg/kg) 15 min prior to the doxorubicin injection (5 mg/kg). All the treatments were given by intraperitoneal route. Both amifostine and doxorubicin were diluted in 0.9% of physiological saline solution immediately before application, according to the manufacturer's instructions and were administered in single doses.

### Histological procedures and histopathological analysis

Before the euthanasia, the rats were anaesthetized with thiopental (Tiopentax; Crisália Produtos Químicos e Farmacêuticos, São Paulo, Brazil); their testes were removed and immersed in Bouin's fixative for 48 hrs. Testicular fragments were processed and Paraplast-Plus^® ^(Sigma-Aldrich Co., St. Louis, MO, USA) embedded. Three μm-thick cross sections (two non-consecutive cross sections from each testis) were stained with Hematoxylin and Eosin or submitted to the Periodic Acid-Schiff (PAS) method and counterstained with Hematoxylin (PAS+H). The PAS+H method was used since it is a histochemical method that assures an efficient identification of spermatid steps [29]. Two hundred tubular sections per animal (one hundred per testis) were randomly analyzed using the Leica QWin V3 (Cambridge, UK) image analysis system and ×20 objective lens. Tubular sections presenting histopathological alterations were scrutinized and recorded using a digital camera connected to a light microscope.

### Tubular frequency according to the different germ cell types

The frequencies of tubular sections containing each germ cell type (spermatogonia, primary spermatocytes, round spermatids and elongated spermatids) were obtained [30-33]. For this aim, fifty PAS-treated seminiferous tubule sections per testis (left and right testes) were randomly examined at ×1000 magnification, totaling 100 tubular sections per animal. The results are expressed in percentage. The identification of germ cell types was performed according to Leblond & Clermont [29].

### Stereological and morphometric analyses

Immediately after testis removal from the scrota, they were weighted and their volumes were obtained by Scherle's method [34]. The volume densities (Vv) of Tubular Lumen (VvTL), Seminiferous Epithelium (VvEp), Lymphatic Space (VvLS) and Interstitial Tissue (VvIT) were obtained. For this purpose, a 25-point integrating eyepiece [35] was coupled to a light microscope. Thirty random fields of right and left cross sections of each testis were analyzed at 125× magnification, totaling 750 points per testis (1500 points per animal).

Tubular diameter and seminiferous epithelium height of 50 random tubular cross-sections per testis were analyzed, using a micrometer eyepiece attached to a light binocular microscope, at 80× magnification. When a section was oblique, only the minor axis of the tubular section was measured [25,26].

### Sperm concentration, motility and morphology

Samples of epididymal cauda fluid were obtained for the analysis of sperm concentration, motility and morphology. All these procedures followed the consensus report published by Seed and colleagues [36].

After properly cleaned, the left epididymis was placed on a Petri dish containing 5 ml of 0.9% saline solution; then, the distal part of epididymis cauda was minced with a razor blade to allow sperm diffusion in the physiological solution for 5 min, under 26-30°C. An aliquot (50 μl) of the sperm homogenate was placed on a slide for motility assessment. Motile (showing any kind of movement) and non-motile (steady) sperm cells were counted within 5 different fields. An hour after the sperm diffusion in saline solution, sperm counts were performed in a hematocytometer chamber (Neubauer Bright Line Improved, 0.100 mm). Morphological analyses of spermatozoa smears of control and experimental rats were also performed. The smears were stained using Shorr/Hematoxylin method and the percentages of normal and abnormal spermatozoa were counted (200 spermatozoa/rat). The determinant abnormal characteristics considered were: a) the shape and size of spermatozoa head, including big or small heads, with lighter or accentuated curvature; b) intermediary pieces defects resulting untied heads; c) defects of tails including short, multiple, folded or broken tails.

### Reproductive competence

Each fifteen 90-day-old males from SC, A, D and AD groups were mated with two normal primiparous females in proestrous. Sperm positive females were killed at the 21^st ^day of pregnancy to access male fertility index (the ratio between the number of live fetuses and the number of mated females) [32].

### Statistical analysis

The morphometric and stereological data were submitted to parametric and non-parametric tests using Jandel Statistical SigmaStat software 2.0. "Standard One Way Anova" parametric and "Anova on Ranks" non-parametric tests were used to respectively evaluate the statistical significance among means or medians from the obtained data. When results showed statistical significance, data were submitted to Student-Newman-Keuls multiple-comparison test. Differences among the groups were considered significant when p < 0.05.

## Results

All rats treated with 400 mg/kg of amifostine (A and AD groups) showed lethargy, shivers and piloerection soon after the treatment. The rats only treated with doxorubicin showed alopecia around ten days after the treatment.

No significant differences were observed regarding the body weight (data not shown) among the different groups (SC, A, D, AD).

### Histopathology of the seminiferous epithelium

**Figure 1:**
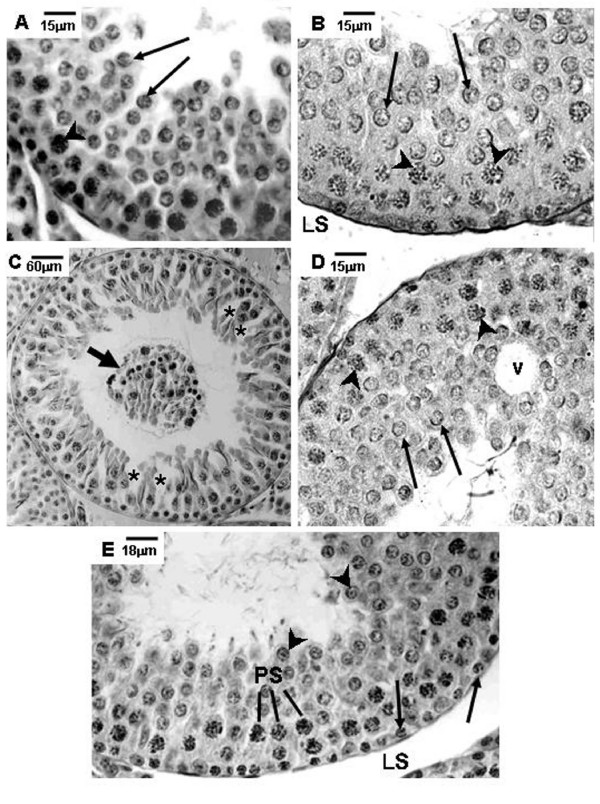
**Photomicrographs of testicular sections of Sham Control, Amifostine, Doxorubicin and Amifostine/Doxorubicin treated rats at different ages**. PAS+H method. Portions of tubular sections of 45-day-old rats. **A and B**: Sham Control (**A**) and Amifostine-treated (**B**) groups showing organized seminiferous epithelium containing various cell types until round spermatids. Lymphatic space (LS); primary spermatocytes (arrowheads); round spermatids (long arrows). **C and D**: Doxorubicin-treated group. Note in **C **the cellular debris and the germinal lineage cells detached from the epithelium into the tubular lumen (short arrow); see also the discontinuous seminiferous epithelium (asterisks). In **D**, observe the vacuole formation (v), the primary spermatocytes (arrowheads) and the round spermatids (long arrows). **E**: Amifostine/Doxorubicin-treated group showing the organized seminiferous epithelium with normal morphology. Spermatogonia (arrows); primary spermatocytes (PS); round spermatids (arrowheads).

**Figure 2:**
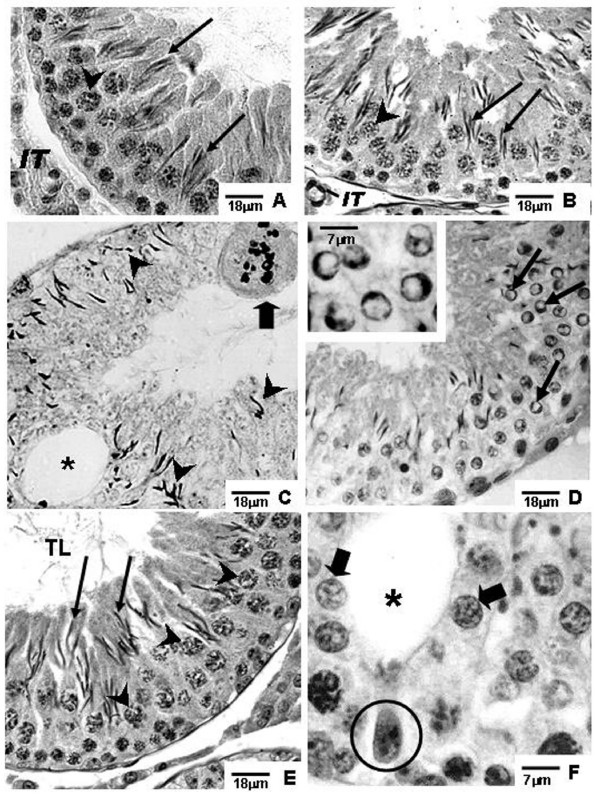
**Photomicrographs of testicular sections of Sham Control, Amifostine, Doxorubicin and Amifostine/Doxorubicin treated rats at different ages**. PAS+H method. Portions of sections of seminiferous tubules of 60-day-old rats. **A and B**: Sham Control (**A**) and Amifostine (**B**) groups. Elongated spermatids (arrows); interstitial tissue (IT); primary spermatocytes (arrowheads). **C and D**: Doxorubicin-treated group displaying damaged seminiferous epithelium. In **C**, an accentuated epithelial depletion and various elongated spermatids, sometimes abnormally located (arrowheads) can be observed; vacuole (asterisk); multinucleated formation in degeneration (thick arrow). In **D**, some spermatids showing outlined condensed and ring-shaped marginal chromatin are observed; this event suggests apoptosis occurrence (thin arrows). **Inset**: detail of round spermatids with ring-shaped marginal chromatin. **E and F**: Amifostine/Doxorubicin-treated group. Note the presence of morphologically normal round (thick arrows) and elongated (long arrows) spermatids, vacuole (asterisk) and Sertoli cell nucleus dislocated from the tubular periphery and with abnormal chromatin condensation (circle); primary spermatocytes (arrowheads); tubular lumen (TL).

**Figure 3:**
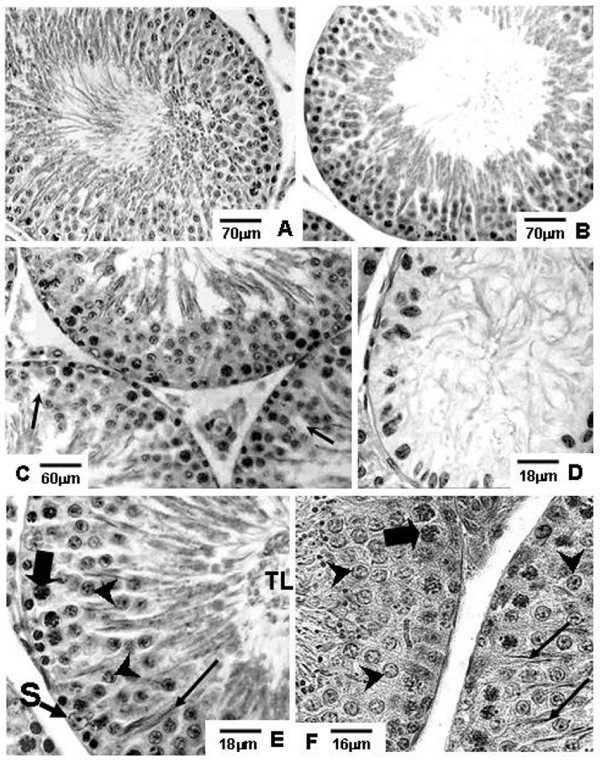
**Photomicrographs of testicular sections of Sham Control, Amifostine, Doxorubicin and Amifostine/Doxorubicin treated rats at different ages**. PAS+H method. Portions of seminiferous tubule sections of 90-day-old rats. **A **and **B**: Sham Control (**A**) and Amifostine (**B**) groups showing normal organization of the seminiferous epithelium. **C **and **D**: Doxorubicin-treated group; in **C**, tubular sections with partial depletion of the seminiferous epithelium are noted; some areas of discontinuity in the germ cell layers can be observed (arrows); in **D**, a tubular section with accentuated cellular depletion and showing Sertolization is shown. **E **and **F**: Amifostine/Doxorubicin-treated group; in both figures, note the concentric and normally organized germ cell layers of the seminiferous epithelium; pachytene spermatocytes (thick arrows); Sertoli cell nucleus (S); round spermatids (arrowheads); elongated spermatids (long arrows); tubular lumen (TL).

Amifostine-treated rats of the A and AD groups showed preserved seminiferous epithelium, with normal morphology, similar to those observed in rats of the Sham control group, at the corresponding ages (Figs. 1A, B, E, 2A, B, E, F, 3A, B, E and 3F).

Different degrees of seminiferous tubule damage were noted in the solely doxorubicin-treated rats according to the age they were killed. Some tubular sections of 45-day-old doxorubicin-treated rats showed disorganized seminiferous epithelium, with discontinuous germ cell layers, as well as sloughed germ cells detached into the tubular lumen (Figs. 1C and 1D). In 60-day-old doxorubicin-treated rats, depletion of seminiferous epithelium, intraepithelial vacuolization and multinucleated formations of round spermatids (Fig. 2C) were the most common alterations noted. Round spermatid nuclei with condensed peripheral chromatin suggesting apoptosis were also observed in tubular sections where the seminiferous epithelium was apparently more preserved (Fig. 2D). On the other hand, rats of 90D subgroup displayed a more preserved seminiferous epithelium than those of the 60D subgroup, although they still presented some morphological damage. Germ cell loss was occasionally observed in this subgroup (Figs. 3C and 3D). Conversely, rat testes submitted to doxorubicin treatment, which were previously-treated with amifostine (AD group), showed normal concentric distribution of germ cells, with organized layers arranged according to the cytological differentiation degree, likewise noted in SC and A rats (Figs. 1E, 2E, F, 3E and 3F). Occasional intraepithelial vacuoles were also found in the seminiferous tubule sections of 60AD rats (Figs. 2E and 2F). Some Sertoli cell nuclei were displaced and showed abnormal condensed chromatin (Fig. 2F) in these rats.

### Stereological analysis: volume densities (Vv)

The volume densities of the seminiferous epithelium, tubular lumen, interstitial tissue and lymphatic space are shown in Table 1.

**Table 1:** Stereological measurements, expressed in percentages, obtained from the testicular components of rats of the SC, A, D and AD groups in three different phases of sexual maturation (Mean ± std. dev.)

Group	Volume Density - Vv (%)			
	VvTL	VvEp	VvIT	VvLS
**45SC**	10.12 ± 1.12	66.64 ± 2.31	8.76 ± 0.89	14.48 ± 2.87
**45A**	9.02 ± 0.80^*c*^	62.38 ± 4.40	7.76 ± 0.78^*a, c*^	20.84 ± 4.52 ^*a*^
**45D**	11.46 ± 1.19 ^*a, b, d*^	65.56 ± 2.09	6.64 ± 0.64 ^*a, b*^	16.34 ± 2.00
**45AD**	9.40 ± 0.54 ^*c*^	64.68 ± 1.29	6.64 ± 0.41^*a*^	19.26 ± 1.86
**60SC**	5.38 ± 0.65	64.32 ± 1.37	9.70 ± 0.44	20.74 ± 1.13
**60A**	7.42 ± 0.47 ^*a, c, d*^	69.30 ± 1.92 ^*a, c*^	9.18 ± 0.28 ^*c*^	14.10 ± 1.98^*a, c*^
**60D**	10.56 ± 1.32 ^*a, b, d*^	58.22 ± 2.24^*a, b, d*^	8.42 ± 0.49^*a, b*^	22.80 ± 2.61^*b, d*^
**60AD**	9.28 ± 0.37 ^*a, b, c*^	70.00 ± 1.52 ^*a, c*^	8.42 ± 0.75 ^*a*^	12.50 ± 1.87^*a, c*^
**90SC**	10.82 ± 0.58	65.48 ± 2.33	9.30 ± 0.36	15.16 ± 1.43
**90A**	11.78 ± 1.07 ^*c, d*^	65.00 ± 3.21^*c, d*^	8.78 ± 0.76 ^*c, d*^	14.42 ± 3.33^*d*^
**90D**	16.40 ± 1.92^*a, b, d*^	57.60 ± 2.35^*a, b, d*^	6.80 ± 0.60^*a, b, d*^	19.20 ± 2.32^*d*^
**90AD**	8.54 ± 0.52^*a, b, c*^	61.70 ± 1.59^*b, c*^	7.94 ± 0.51 ^*a, b, c*^	22.00 ± 2.05 ^*a, b, c*^

**Volume Densities (Vv)**: Tubular Lumen (**VvTL**); Seminiferous Epithelium (**VvEp**);

Interstitial Tissue (**VvIT**); Lymphatic Space (**VvLS**)

***P *< 0.05 **(significant): a = significant when compared to SC; b = significant when

compared to A; c = significant when compared to D; d = significant when compared

to AD

The values of seminiferous epithelium volume density (VvEp) were lower in the 60- and 90-day-old solely doxorubicin treated rats (60D and 90D subgroups) than in rats of the other corresponding subgroups), including those amifostine/doxorubicin treated (AD group). Conversely, there was a reduction of VvEp in 90-day-old amifostine/doxorubicin treated rats (90AD subgroup) in comparison with those of the 90A subgroup; however, no significant differences were observed regarding this parameter when 90AD and 90SC subgroups were compared.

The lymphatic space volume density (VvLS) was significantly reduced in 60-day-old amifostine-treated subgroups in comparison to the corresponding SC and D subgroups; however, the rats of the 90AD subgroup showed an increase of this parameter in comparison to all other subgroups of the same age (90SC, 90A and 90D).

### Morphometric testicular analysis

**Figure 4:**
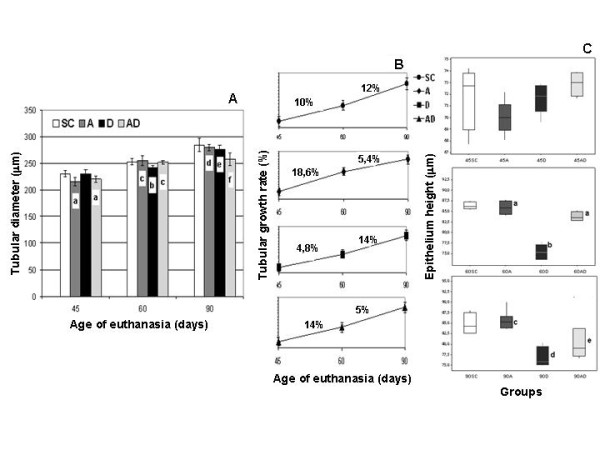
**Seminiferous tubule diameter morphometry in rats of the SC (Sham Control), A (Amifostine), D (Doxorubicin) and AD (Amifostine/Doxorubicin) groups at the 3 ages studied**. Seminiferous tubule diameter (**4A**) and the growth rates (**4B**) observed in the different groups. **4C**: Box plots illustrating the median of the seminiferous epithelium height. The median for each dataset is indicated by the line in the boxes which in themselves contain 50% of the data. The upper and lower hinges of the boxes indicate the 25-75 percentiles. Values within 1.5 times the interquartil range are indicated by the lines. Mean values +/- standard deviation; P < 0.05 (a, b, c, d, e, f). **Labels **- **4A: a **= A and AD < SC and D; **b **= D < SC, A and AD; **c **= A and AD > D; **d **= A > AD; **e **= D > AD;**f **= AD < SC, A and D. **4C**: **a **= A and AD > D; **b **= D < SC, A and AD; **c **= A > D and AD; **d **= D < SC, A and AD; **e **= AD < SC and A.

No significant differences were observed regarding the testis weight and total testicular volume (data not shown) among the different groups (SC, A, D, and AD). A gradual increase of seminiferous tubule diameter occurred from 45 to 90 days of age in all groups (Fig. 4A); however, this growth rate was significantly reduced in amifostine-treated rats (A and AD groups) from 60 to 90 days of age, when they were compared to the SC and D groups, as shown in the Fig. 4B. Nevertheless, from 45 to 60 days of age, rats of the D group presented a reduced growth rate of the tubular diameter in comparison to all the other groups.

A considerable reduction of the seminiferous epithelium height (Fig. 4C) was observed in the 60D and 90D subgroups when they were compared to the other corresponding subgroups (60SC, 90SC; 60A, 90A; 60AD and 90AD); a reduction of this parameter was also observed in the rats of the 60AD and 90AD subgroups in comparison to those from the Sham Control and Amifostine subgroups at same ages.

### Tubular frequency according to the different germ cell types (Table 2)

**Table 2:** Frequencies of the different types of germinal lineage cells per 100 tubular sections examined in rat testes of the SC, A, D and AD groups, in three different phases of sexual maturation (Mean ± std. dev.)

Group	Cell Type (%)		
	GA	GIn	GB
**45SC**	72.40 ± 3.05 ^b, c, d^	42.00 ± 4.95 ^b, c, d^	54.00 ± 4.95^b, c, d^
**45A**	75.80 ± 1.92^a, c, d^	51.20 ± 4.02 ^a, c^	29.60 ± 4.15^a^
**45D**	65.80 ± 2.49^a, b^	33.80 ± 3.56^a, b, d^	24.80 ± 3.96^a^
**45AD**	66.80 ± 1.48^a, b^	56.00 ± 4.74^a, c^	27.20 ± 6.27^a^
**60SC**	85.20 ± 3.56^c^	60.00 ± 5.52^b, c^	67.20 ± 5.76^c, d^
**60A**	88.00 ± 2.23^c, d^	74.80 ± 3.34^a, c, d^	66.00 ± 5.00^c, d^
**60D**	77.00 ± 2.55^a, b, d^	49.00 ± 3.97^a, b, d^	39.40 ± 7.02^a, b, d^
**60AD**	83.20 ± 3.03^b, c^	59.60 ± 5.94^b, c^	48.60 ± 4.33^a, b, c^
**90SC**	97.20 ± 1.30^b, c, d^	48.40 ± 4.98^b, d^	53.00 ± 3.93^b, c, d^
**90A**	100.00 ± 0.00^a, c, d^	62.60 ± 3.64^a, c, d^	43.40 ± 3.05^a, b, d^
**90D**	89.80 ± 1.92^a, b, d^	44.80 ± 3.03^b^	27.00 ± 0.94^a, b, d^
**90AD**	93.20 ± 2.77^a, b, c^	41.80 ± 2.58^a, b^	35.68 ± 1.28^a, b, c^
	**PS**	**RS**	**ES**
**45SC**	99.80 ± 1.05	72.60 ± 3.64	99.00 ± 1.00^b, d^
**45A**	100.00 ± 1.00	73.40 ± 2.70	84.20 ± 5.26^a, c, d^
**45D**	99.80 ± 1.20	72.80 ± 7.91	98.00 ± 2.00^b, d^
**45AD**	99.80 ± 1.05	78.40 ± 1.81	74.00 ± 11.95^a, b, c^
**60SC**	100.00 ± 0.03	83.60 ± 3.57^c, d^	98.40 ± 2.07
**60A**	100.00 ± 0.02	82.40 ± 1.94^c, d^	99.00 ± 1.22
**60D**	100.00 ± 0.03	73.60 ± 3.43^a, b^	97.00 ± 4.58
**60AD**	99.80 ± 0.03	77.20 ± 4.76^a, b^	99.20 ± 1.09
**90SC**	100.00 ± 0.01	85.60 ± 2.19^c^	99.90 ± 0.44
**90A**	100.00 ± 0.00	85.80 ± 1.92^c^	100.00 ± 0.00
**90D**	99.60 ± 0.50	71.60 ± 2.40^a, b, d^	98.60 ± 1.34
**90AD**	99.40 ± 0.80	82.20 ± 4.14^c^	99.40 ± 0.89

**GA**: Type A spermatogonia; **GIn**: Intermediate spermatogonia; **GB**: Type B spermatogonia; **PS**: Primary spermatocyte; **RS**: Round spermatids; **ES**: Elongated spermatids

***P *< 0.05 **(significant): a = significant when compared to SC; b = significant when compared to A; c = significant when compared to D; d = significant when compared to AD

In the 45D subgroup, the frequency of seminiferous tubule sections containing intermediate spermatogonia (GIn) was lower in comparison to those from other subgroups at same age (45SC, 45A and 45AD). On the contrary, no significant differences were observed between 45D and 45AD subgroups regarding the frequencies of tubular sections containing types A and B spermatogonia (GA and GB). Besides, this parameter was reduced in the 45D and 45AD subgroups in comparison with those observed in the 45SC and/or 45A subgroups.

Conversely, sixty-day old rats of D group showed a significant reduction of the frequencies of seminiferous tubule sections containing the three types of spermatogonia (GA, GIn and GB) when compared to all other subgroups at the same age. In addition, no significant reductions of the number of tubular sections containing GA and GIn were observed in testes of 60AD rats in comparison to 60SC rats.

On the other hand, rats of the 90D subgroup showed significantly reduced frequencies of tubular sections containing GA and GB, in comparison to the other subgroups (including the 90AD group), except for GIn spermatogonia type. In addition, rats of 90AD subgroup showed a lower frequency of tubular sections containing GA, GIn and GB than those of the 90SC and 90A subgroups.

The frequency of tubular sections containing round spermatids (RS) decreased in rats of the 90D subgroup in comparison to all other subgroups at the corresponding ages, including the AD group. In spite of the same reduction was observed in the 60D subgroup, it was not significantly lower when compared to the 60AD subgroup. Nevertheless, 45-day-old amifostine-treated rats (45A and 45AD subgroups) showed a reduction of frequency of tubular sections with elongated spermatids (ES) in comparison to the rats of 45SC and 45D subgroups.

### Concentration, motility and morphology of spermatozoa in the epididymal fluid

**Figure 5:**
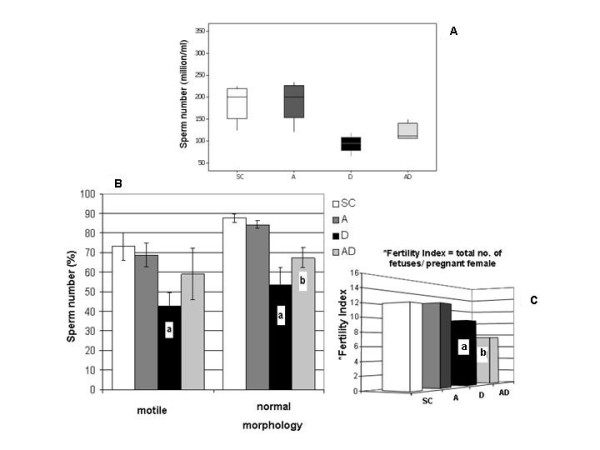
**Graphics illustrating the sperm parameters and the fertility index of 90-day-old rats, obtained from the SC (Sham Control), A (Amifostine), D (Doxorubicin) and AD (Amifostine/Doxorubicin) groups**. **5A**: Box plots showing the median for sperm concentration in each group indicated by the line in the boxes which in themselves contain 50% of the data. The upper and lower hinges of the boxes indicate the 25-75 percentiles. Values within 1.5 times the interquartil range are indicated by the lines. **5B**: Percentage of sperm that displayed any type of movement and normal morphology; **5C**: Fertility index. Mean values +/- standard deviation; P < 0.05 (a and b). **Labels **- **5A: a **= D < SC, A and AD; **b **= AD < SC and A. **5B**: **a **= D < SC, A and AD; **b **= AD < SC and A. **5C**: **a **= D < SC and A; **b **= AD < SC, A and D.

In the 90AD subgroup, all sperm parameters analyzed (sperm number, motility and normal morphology percentages - Figs. 5A and 5B) were significantly increased when compared to those corresponding to the 90D subgroup. However, although sperm concentration and normal morphology observed in amifostine/doxorubicin-treated rats showed an improvement in comparison to those obtained from the D group, it was still reduced when compared to the rats of the SC and A groups.

### Reproductive competence

The number of fetuses obtained from the females mated with rats from the 90D and 90AD subgroups was decreased when compared to the number of fetuses observed in the females mated with rats from the 90SC and 90A subgroups (Fig. 5C). Interestingly, the 90AD subgroup had a lower fertility index when compared to the 90D subgroup as well.

## Discussion

The results of this study showed that amifostine promotes an improvement of some testicular and seminal parameters, suggesting that amifostine reduced the doxorubicin toxicity on the male gonad. However, the analysis of the data, which were obtained after the elapsed time of sixty days (from treatment to the euthanasia of 90D and 90AD rats), showed that the amifostine pre-treatment did not guarantee fertility improvement of the sequentially doxorubicin-treated animals, although some reduction of testis damage and an improvement of the sperm concentration, motility and morphology have been observed. The protective capacity of amifostine over some other tissues has already been proven to be effective against the deleterious action of gamma irradiation, taxanes, antracyclines and platinum derivatives [5,16,17,21,23,37].

Both doxorubicin and amifostine doses chosen to be used in this study were based on previous findings. Doxorubicin is usually administered to adult rats in doses up to 21 mg/kg [38,39]. In rodents, the single dose of 5 mg/kg of body weight provokes disruption on spermatogenic cells maturation, epididymis sperm concentration reduction, alterations of spermatogonia DNA and sperm morphology [40]. The aim of this study was to verify if a single dose of amifostine could reduce the long-term side effects on the seminiferous epithelium of sexually immature and adult rats, provoked by a unique cytotoxic dose of doxorubicin administered in the prepuberty. On the same way, the single dose of 400 mg/Kg of amifostine was selected due to its testicular minimal toxicity which could be neglected, as described by Meistrich and co-workers in murine assays [41]; on the other hand, single higher doses or multiple lower doses have been referred to be toxic to the stem spermatogonia, diminishing their survival [15,41].

Few studies on the association of amifostine and doxorubicin in cancer therapy have been recently carried out; some of them produced contradictory results regarding the protection of the bone marrow and of cardiac and testicular tissues [25-28]. Therefore, amifostine potential protection against doxorubicin testicular toxicity seems to be a very delicate issue. Thus, we decided to carry out a detailed morphometric and stereological analyses of rat testes which constitute important unbiased tools to study biological tissue damages.

The alteration of the measurements of testicular volume and weight, for example, suggests injury of the gonad. In addition, parameters such as tubular diameter, seminiferous epithelium height and volume densities of tubular lumen and seminiferous epithelium can also give information about the testicular damage degree as a consequence of germ cell death. França and Russell [42] have mentioned that when a massive germ cell loss occurs, it is followed by a sharp decline in testicular morphometric parameters. In general, germ cell death caused by anticancer drugs, including doxorubicin [43,44], culminates with a reduction of morphometric parameters [24,33]. However, our data suggest that germ cell loss is not necessarily associated with the reduction of these parameters since the occurrence of other phenomenon can interfere in the final establishment of the testis weight [24]. In fact, alterations of testicular weight were not observed in doxorubicin and amifostine-treated rats; since germ cell loss was noticed, the unaltered weight might be a consequence of a lymphatic and/or interstitial edema that counterbalance germ cell death and the organ weight. Interstitial edema is a testicular injury and a direct consequence of endothelial layer disruption, which liberates fluids from blood flow into interstitial tissue. Thus, testicular interstitial fluid volume can be increased by a variety of factors including testosterone level alterations [45] and toxic exposure [24,46,47]. Indeed, doxorubicin causes endothelial dysfunction and edema, as secondary effects of oxidative stress in the vascular wall. The vascular endothelium plays a fundamental role in maintenance of organ function by forming a barrier regulating water and solute distribution between blood and tissues; however, this fluid control can be deregulated by oxidative stress [48], resulting in movement of water and proteins from the vascular system into tissues and compromising organ function. In fact, in 90-day-old rats of the AD group, for example, the lymphatic capillary volume density was conspicuously increased when compared to the other corresponding groups in the same age. Future studies must be carried out to clarify the effects of amifostine on testis interstitial tissue, and the impact of the interaction of the amifostine and doxorubicin on the hormonal regulation of the male reproductive system, the endothelial dysfunction and testicular edema occurrence, as well as their roles on the morphophysiology of the interstitial tissue.

In spite of the adverse effects observed on the lymphatic space volume density, it was noted a distinctive reduction of the seminiferous epithelium damage in the 60-day-old rats treated with amifostine/doxorubicin, in comparison to those solely doxorubicin-treated. Indeed, amifostine seems to reduce the damage and the germ cell loss caused by the acute doxorubicin treatment and this can be confirmed when the present data related to the epithelium height and volume density from the 60- and 90-day-old doxorubicin-treated rats previously treated or not with amifostine were compared. The analysis of the spermatogonia frequencies also corroborates this idea. The depletion of seminiferous epithelium and the consequent decrease of morphometric and stereological measurements caused by cytotoxic agents were shown by Meistrich [8] and were confirmed in our report. As previously referred, a significant reduction of the frequency of tubules containing at least one type of differentiated spermatogonia (types A, Intermediate and B) occurred in the doxorubicin groups, in comparison to the amifostine-pretreated groups. Although spermatogonia are not the only cell type of the seminiferous epithelium under continuous division, their localization in the basal portion of the epithelium make them more vulnerable to the action of cytotoxic drugs [7,14,33].

Considering that, in rats, the complete seminiferous epithelium cycle lasts 12 days [49,50], we can suppose that, according to the elapsed time between treatment and the euthanasia of the animals applied in our study (15, 30 or 60 days), the cells generated from the target spermatogonia were: pachytene spermatocytes at 15 days; round spermatids at 30 days and mature sperm that reach the epididymis cauda at 60 days [51]. Even though the total number of each cell type was not quantified in the current study, we can assume based on the results that there was higher cell loss from the seminiferous tubules in the solely doxorubicin-treated rats in comparison to those which were previously amifostine-treated. On the other hand, it is plausible to consider that the progression of cellular maturation could be deregulated by amifostine treatment based on the alterations of the frequencies of tubular sections containing intermediate spermatogonia and spermatids, which were observed in the 45-day-old rats from amifostine-treated subgroups (45A and 45AD). We believe that such alteration might have occurred due to one of the amifostine protective mechanisms of DNA interaction, i.e., producing its stabilization and inhibiting nuclear proteins, such as topoisomerase II [52] and DNA-polimerase [53], which were involved in cell division. Thus, while intercalated with the DNA, amifostine prevents doxorubicin linkage with cell DNA [20]. The decreased frequency of tubular sections with spermatids observed in 45AD subgroup, even when compared to 45A subgroup, might have occurred as a consequence of interference on the cell cycle caused by amifostine-doxorubicin association [37]. Nevertheless, this effect was reversible since it was only detected in 45-day-old rats.

It is important to emphasize that two studies carried out by a same group have shown amifostine toxicity to 6-day-old rat germ cells when associated with doxorubicin [26,28]. In their most recent study they have suggested that the treatment with amifostine (200 mg/kg) prior to doxorubicin (3 mg/kg b.w.) did not provide any protection against apoptosis induced in rat stem spermatogonia of 6-day-old rats, whereas 16- and 24-day-old animals did not show increase of cell death caused by doxorubicin [28]. Considering the low dose used in the aforementioned study, it is probable that the seminiferous epithelium of 16-day-old rats is more resistant to the doxorubicin than that of the 6-day-old rats, since the hematotesticular barrier begins to be established at the age of 15 days, according to Schulze [54]. As previously demonstrated by our group, 30-day-old rats did not show considerable toxicity after treatment with a 400 mg/kg single dose of amifostine [24]. On the other hand, the chosen dose of doxorubicin used in the present experiment was unable to cause mortality or reduction of body weight in rats at all studied ages, although morphological damages of seminiferous epithelium have occurred as a consequence of its cytotoxicity.

Doxorubicin is known to produce apoptosis [28,43] on dividing cells, as etoposide [33]. Our histological findings suggest that doxorubicin cytotoxicity was responsible for producing the frequently observed apoptotic round spermatids and multinucleated apoptotic cells. The intercalation of doxorubicin in the germ cell DNA during division is considered to be the principal cause of cellular death induction in the seminiferous epithelium [9,43]. Even escaping from death, the cells affected by doxorubicin can still have its genome damaged, provoking further impairments on cell progression [55].

Confirming our histological findings, sperm analysis from the epididymal fluid also suggests that amifostine could confer partial protection for cell survival. The significantly higher sperm concentration obtained in prior-amifostine-treated rats leads us to believe that there were a higher number of resistant spermatogonia in these animals. However, reproductive competence results alerted us about the level of the protection conferred by amifostine against the potential damage produced on sperm membrane and DNA integrity after doxorubicin exposure. Under the experimental conditions used here, amifostine conferred some germ cell protection against doxorubicin probably by preventing apoptosis of healthy and genetically damaged spermatogonia. If spermatogonia with harmed DNA survived, it is possible that they produced sperm with damaged DNA, although good sperm motility and morphology have been observed. Thus, the sperm with damaged DNA would be able to compete with normal sperm and produce abnormal embryos, what leads to the conclusion that such improvement in spermatogonia survival is disadvantageous for reproduction and embryo development. These subjects are under investigation.

## Conclusions

Nowadays, the impairment of infertility remains a struggle for surviving patients who have undergone chemotherapy treatment, especially during childhood. As demonstrated in the current study, the testicular cytotoxicity caused by doxorubicin provokes serious germ cell depletion in the seminiferous epithelium of prepubertal rats. Our results have shown a reduction of seminiferous epithelium damage caused by doxorubicin and an improvement of the epididymal fluid sperm parameters in previously amifostine-treated rats; thus, they have pointed out for a partial protection of germ epithelium, although the aspects related to the combined administration of amifostine and doxorubicin and its impact on the germ cell genome components must be clarified. Spermatogonia survival promoted by amifostine was disadvantageous when doxorubicin was taken together, leading to reduced reproductive outcome. In this scope, sperm DNA integrity and its future contribution to the subsequent generation health might be a concern. Thenceforth, more detailed studies are necessary to investigate how they interact when concomitantly administered. In order to explore the potential cytoprotective benefits of amifostine, the protocols of administration and the age of treatment must be carefully considered.

## Competing interests

The authors declare that they have no competing interests.

## Authors' contributions

SMM coordinated all steps of the study. VV carried out all the experimental procedures, data and photomicrographs acquisition. VV, ESC and SMM examined and selected the images. All authors participated in the design and writing of this study; each one of them also read and approved the final manuscript.
